# Pinhole Zone Plate Lens for Ultrasound Focusing

**DOI:** 10.3390/s17071690

**Published:** 2017-07-22

**Authors:** Constanza Rubio, José Miguel Fuster, Sergio Castiñeira-Ibáñez, Antonio Uris, Francisco Belmar, Pilar Candelas

**Affiliations:** 1 Centro de Tecnologías Físicas, Universitat Politècnica de València, Camino de Vera s/n, 46022 Valencia, Spain; crubiom@fis.upv.es (C.R.); auris@fis.upv.es (A.U.); fbelmar@fis.upv.es (F.B.); 2 Departamento de Comunicaciones, Universitat Politècnica de València, Camino de Vera s/n, 46022 Valencia, Spain; jfuster@dcom.upv.es; 3 Departamento de Ingeniería Electrónica, Universitat de València, Avd. de la Universitat s/n, 46100 Burjassot, Valencia, Spain; casiser@uv.es

**Keywords:** ultrasonic lens, Fresnel lens, pinhole

## Abstract

The focusing capabilities of a pinhole zone plate lens are presented and compared with those of a conventional Fresnel zone plate lens. The focusing properties are examined both experimentally and numerically. The results confirm that a pinhole zone plate lens can be an alternative to a Fresnel lens. A smooth filtering effect is created in pinhole zone plate lenses, giving rise to a reduction of the side lobes around the principal focus associated with the conventional Fresnel zone plate lens. The manufacturing technique of the pinhole zone plate lens allows the designing and constructing of lenses for different focal lengths quickly and economically and without the need to drill new plates.

## 1. Introduction

Beam forming and focusing can be achieved by devices based on refraction or diffraction phenomena. Focusing certain electromagnetic waves, such as soft X-rays and extreme ultraviolet radiation, by means of refracting optics devices has important limitations due to absorption. Diffractive devices are used to improve focusing, and the Fresnel zone plate (FZP) lens is one of the candidates. FZP consists of alternating opaque and transparent rings of certain radius and width in such a way as to cause constructive interference at the focus. However, spatial resolution is limited and depends on the width of the outer rings and it is difficult to acquire high quality patterns. To overcome these limitations, Kipp et al. [1] proposed a diffractive optical element called photon sieve, which is essentially a FZP in which transparent rings are replaced by a great number of pinholes. The holes can be distributed randomly or regularly in the transparent zone. Each pinhole of the photon sieve interferes constructively at the focal point downstream.

The importance of wave focusing is not only significant in the field of electromagnetic waves, but also extends to the acoustic field. Due to the potential applications of acoustic focusing in areas such as non-destructive evaluation or medical treatments, it has become a hot research topic.

Inspired by the studies on extraordinary optical transmission through subwavelength aperture arrays, research has been extended to acoustic waves. Sound transmission through narrow slits and perforated plates has attracted much scientific interest throughout the last 10 years, both theoretically and experimentally [2,3,4,5,6,7]. Numerous studies have been centered on acoustic collimation and subwavelength imaging achieved by periodic arrays of subwavelength apertures [8,9,10,11,12,13,14,15,16].

Like in its optical counterpart, to achieve acoustic focusing through constructive interference of diffracted fields, planar diffraction FZPs have been developed. For instance, Moleron et al. [17] and Li et al. [18] developed acoustic Fresnel lenses in air by coiling up space. Calvo et al. [19] fabricated and characterized an underwater acoustic FZP lens with alternating transparent and opaque zones made of soft silicone rubber.

In this work, a novel improved design of a FZP lens is reported. It is shown that replacing the transparent rings of a FZP by a distribution of holes can be used for ultrasound focusing, resulting in a pinhole zone plate (PZP) lens. A manufacturing technique of the PZP lens from an air-solid phononic plate is shown. The advantage of PZP lenses and their manufacturing technique is that they can be easily manufactured to match the focal point at a desired frequency. By comparing them against conventional FZPs, it will be shown that the PZP lens achieves a reduction of side lobes over the FZP lens.

## 2. Materials and Methods

In order to analyze and compare Franhoufer diffraction of FZPs and PZPs lenses, underwater ultrasound experiments have been performed. Both lenses were made using brass plates with 0.350 m in width and 0. 450 m in length (ρ = 7890 kg/m^3^, c_l_ = 5670 m/s, c_t_ = 3230 m/s ) and 0.002 m thickness, immersed in water (ρ = 1000 kg/m^3^, c_l_ = 1480 m/s).

Taking into account that FZPs are diffractive lenses that consist of a number of radially symmetric rings called Fresnel zones, which alternate between opaque and transparent, the waves transmitted by the transparent rings interfere constructively at the focus. r_n_ is the radial distance to the center of the *n*th transparent ring, given by

r_n_ = (2·n·f·λ + n^2^·λ^2^)^1/2^, (1)

being n = 1, 2, …, N, where N is the total number of rings, f is the focal length and λ is the wavelength. The width, w, of each ring is given by

w = λ·f/(2r_n_), (2)

**Figure 1:**
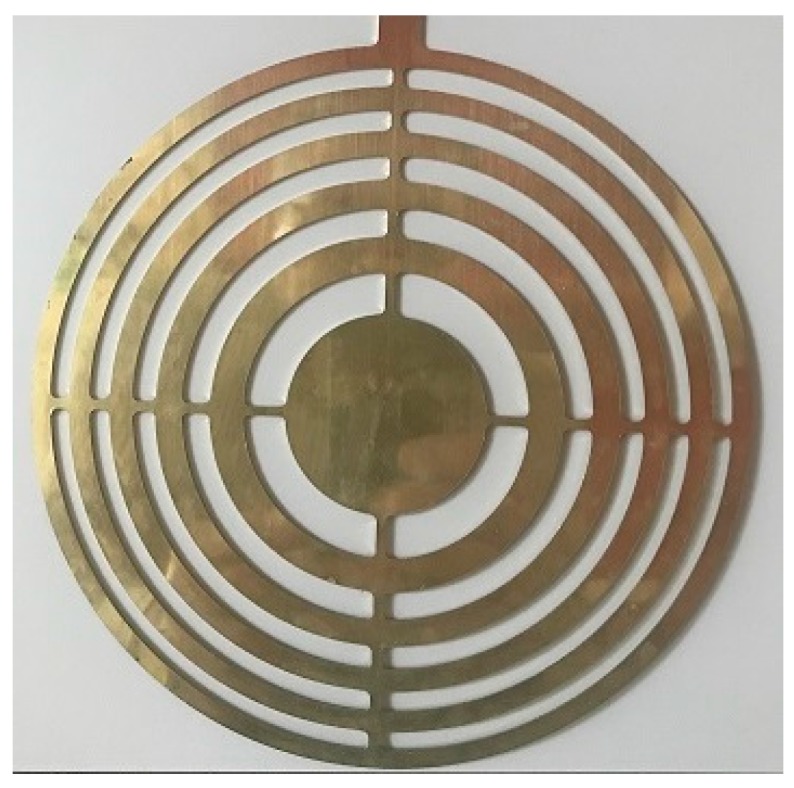
Fresnel zone plate lens considered.

The FZP geometry considered throughout the experiments has 11 rings with the outermost ring radius of 0.0877 m. The lens was designed for a working frequency of 200 kHz and a focal length of 0.07 m. From Equations (1) and (2), the manufacture of the FZP was carried out using a numerical control milling machine. Figure 1 shows a picture of the FZP lens considered in this work.

**Figure 2:**
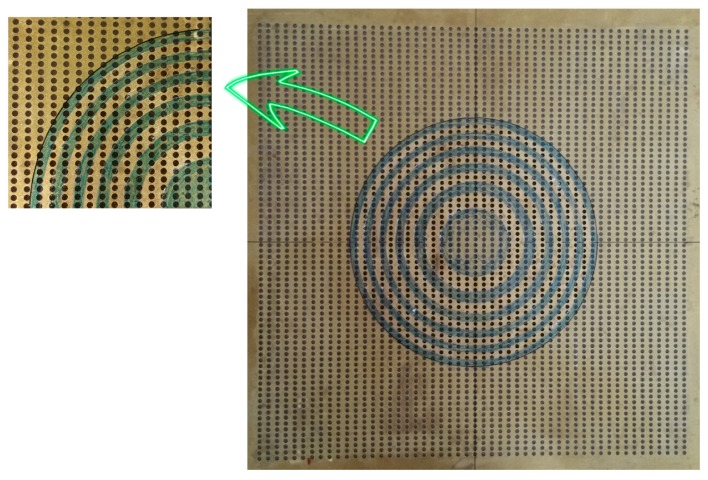
Pinhole zone plate lens considered.

To construct the PZP, in the first step, an air-solid phononic plate was fabricated to restrict the ultrasound wave propagation [20]. A perforated plate with a periodically square distribution of circular holes—0.003 m in diameter and with a unit cell period of 0.006 m—was fabricated. Because air restricts ultrasonic wave propagation, an air–solid phononic plate was built. The fabrication of the air–solid phonic plate was carried out by sticking, carefully, a propylene self-adhesive film (20 μm thick and ρ = 900 kg/m^3^) around the plate, taking care that undesired air bubbles did not appear while the film was adhered. In this way, the air contained in the holes prevented ultrasonic wave propagation, creating opaque zones. To construct the lens, the film was carefully cut and removed from the transparent rings, leaving only the film on the opaque rings. The radius used for these rings are the same as those from the FZP lens. Thus, using a single drilled plate, lenses with different focal lengths can be constructed. Figure 2 shows a picture of the pinhole zone plate lens considered.

**Figure 3:**
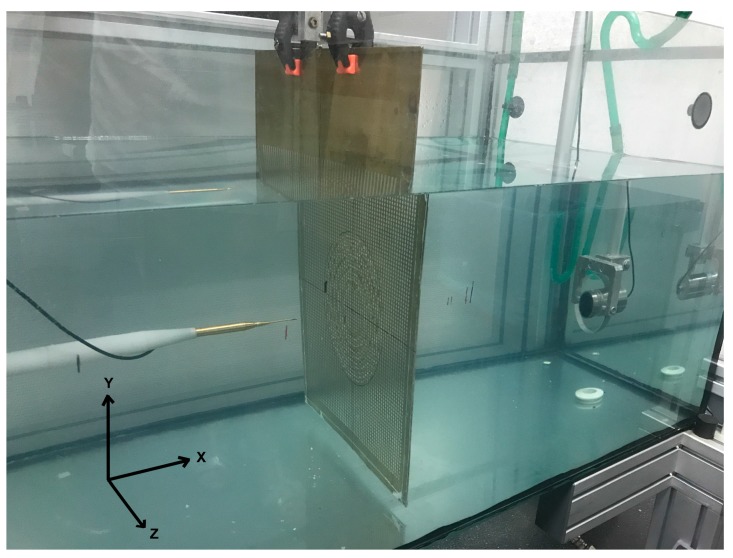
Experimental set-up.

The experimental set-up is based on the ultrasonic immersion transmission technique. The assembly was placed in a water tank. An automated positioning system built around a water tank was used to align and position the hydrophone through a 3D grid of measurement points located at any trajectory inside the tank. The coordinate system used and the experimental set-up are shown in Figure 3. The coordinate origin was located in the center of the lens. A plane inmersion piston transducer (Imasonic, Les Savourots, France) with a center frequency of 250 kHz and with an active diameter of 0.032 m was employed as an emitter and a Polyvinylidene fluoride (PVDF) needle hydrophone (model HPM1/1, Precision Acoustics Ltd., Dorchester, UK) with a diameter of 1.5 mm and with ±4 dB bandwidth spanning from 200 kHz to 15 MHz was employed as a receiver. The pulses launched by the emitter piston transducer were detected by the hydrophone and acquired by the pulser/receiver, post-amplified and digitalized by a digital PC oscilloscope (Picoscope model 3224, Pico Technology, St Neots, UK). Time domain data were finally analyzed after averaging 100 different measurements. Scanning was done using the automated positioning system with a spatial resolution of 1 × 1 mm^2^. The scan area was collinear with the center-line plane of the piston transducer and the sample lenses (XZ plane). 

To verify the focusing properties of the pinhole zone plate lens, the commercial finite element method software COMSOL Multiphysics was used to evaluate the lens. The brass used to construct the lens was modeled as a rigid solid.

## 3. Results

**Figure 4:**
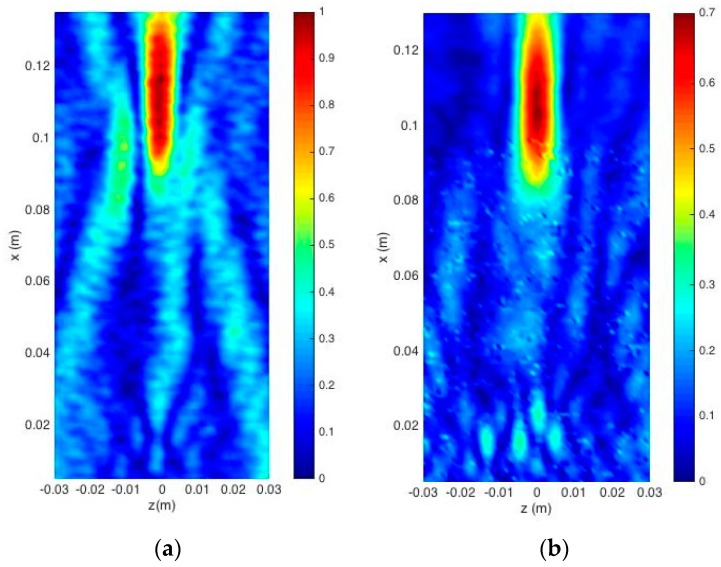
Measured normalized pressure amplitude $\left|p\right|/{\left|p\right|}_{\max}$ at 200 kHz in a XZ plane collinear with center-line plane of the piston transducer and sample lenses. (**a**) Fresnel zone plate (FZP) lens; (**b**) Pinhole zone plate (PZP) lens.

**Figure 5:**
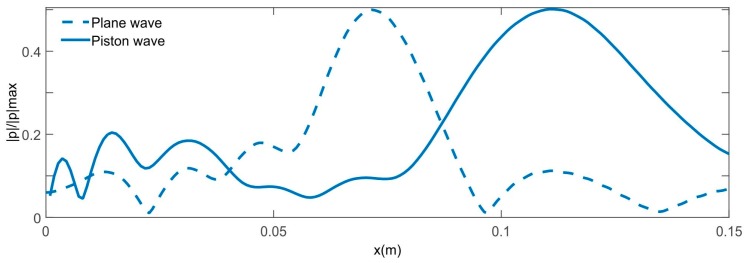
Simulated intensity fields of the normalized acoustic pressure distribution along the x-axis at z = 0 of the Fresnel zone plate lens using plane wave and piston wave.

**Figure 6:**
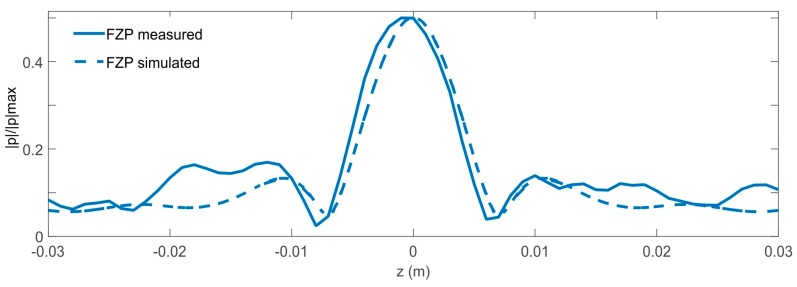
Comparison of the simulated and measured intensity fields of the normalized acoustic pressure amplitude along the z-axis at x = 0.104 m of Fresnel zone plane lens (FZP).

Measurement results of the normalized acoustic pressure amplitude $\left|p\right|/{\left|p\right|}_{\max}$ at 200 kHz had been carried out by means the procedure described in the Materials and Method section, which are plotted in Figure 4 for both lenses. The values of pressure amplitude are normalized by the maximum pressure amplitude of the received wave for both measured lenses. Wave focusing is clearly observed, but the focal lengths do not match with those initially considered. The lenses were designed for a focal length of 0.07 m and experimental focal lengths of 0.104 m were obtained. This is due to the fact that Equations (1) and (2) were obtained for the plane wave case. Since the transducer is a piston, a plane wave is not achieved. This causes the experimental focal length to not match with that used in the lens design [19]. Figure 5 shows a comparison of the normalized acoustic pressure distribution along the x-axis at z = 0 of the FZP lens calculated with COMSOL and using plane wave and piston wave excitation. It is observed that, when replacing the plane wave by a piston wave, the focus is displaced from 0.07 m to 0.1 m. In order to verify the model used in COMSOL, Figure 6 shows a comparison of the acoustic pressure normalized amplitude along the z axis at x = 0.104 m simulated and measured for the FZP lens and using piston wave instead of plane wave. A good agreement between numerical and experimental results is observed.

**Figure 7:**
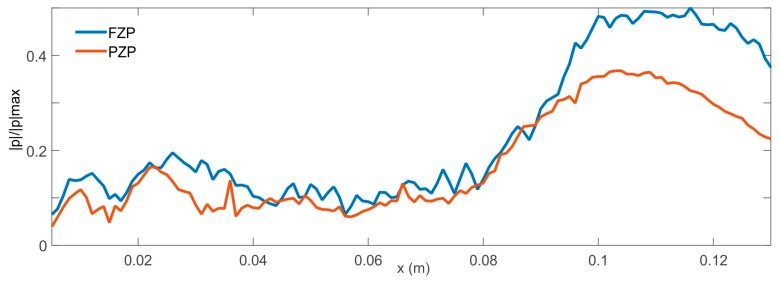
Measured intensity fields of the normalized acoustic pressure amplitude along the x-axis at z = 0 of Fresnel zone plane lens (FZP) and pinhole zone plate lens (PZP).

**Figure 8:**
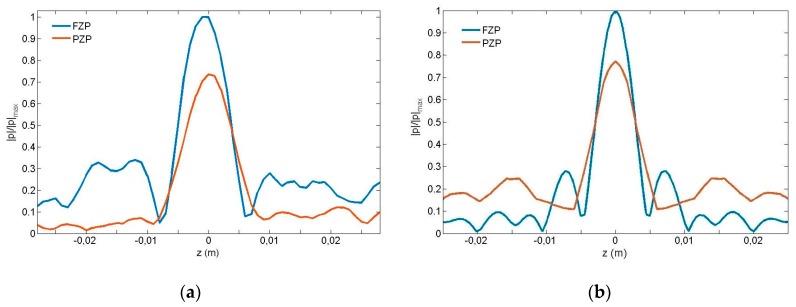
Measured (**a**) and simulated (**b**) intensity distributions along z-axis at the focal point in Fresnel zone plate (FZP) lens and pinhole zone plate (PZP) lens.

The measured distributions of the normalized acoustic pressure amplitude along x-axis at z = 0 of both FZP and PZP lenses are shown in Figure 7. As can be observed from the figure, the sizes for both focal points are comparable. A comparison of focal intensities for FZP and PZP lenses reveals that the transmitted wave intensity is higher in the FZP case than in the PZP one. This is understandable since the total field at the focus is the sum of the fields coming from the individual openings, so it could increase the intensity at the focus in the PZP lens by increasing the hole filling fraction, that is, increasing the diameter of the holes or reducing the unit cell period. Figure 8a,b show the measured and simulated intensity distributions along z-axis at the focal point. It should be noted that due to calculation capacity, the simulated results have been obtained using plane wave excitation because the degrees of freedom in the case of the PZP lens are greater than 20 × 10^6^. Figure 8a shows a 6 dB sidelob reduction on the measured PZP intensity distribution from its FZP counterpart. This experimental side lobes reduction does not match simulations results, but this is due to the fact that plane wave excitation has been used in simulations because of calculation restrains. The side lobe reduction observed in the experimental results is due to the fact that the size of the transparent zones decreases from inside to outside. In the case of PZP lenses, this effect is more pronounced because the outer rings have a smaller width and there is a smaller number of holes, achieving a smooth filtering effect and consequently reducing the side lobes around the principal focus [1]. This reduction of lateral side lobes improves resolution.

## 4. Conclusions

The focusing performance of a PZP has been demonstrated experimentally and numerically. The experimental results are in agreement with the simulated ones. These results have been compared with a traditional FZP lens. In the particular case analyzed, it was observed that focal intensities are higher in the FZP case than in PZP one, due to the fact that total field at the focus is the sum of the fields coming from the individual holes. To increase the intensity at the focus in the PZP lens, it will be necessary to increase the number of holes. Due to the lens design with pinholes, a smooth filtering effect is created and a decrease of the side lobes around the principal focus associated with the conventional FZP lens is achieved. An optimum hole distribution must be implemented in further research to increase the resolution of the PZP lens by increasing the supression of side lobes. On the other hand, the manufacturing technique of the PZP lens allows designing and constructing lenses for different focal lengths without the need to drill new plates.
